# Community-based interventions as an effective program for leishmaniasis treatment: a duty to act

**DOI:** 10.3205/dgkh000441

**Published:** 2023-07-06

**Authors:** Nader Aghakhani, Mehdi Azami, Saeid Amini Rarani

**Affiliations:** 1 Food and Beverages Safety Research Center, Urmia University of Medical Sciences, Urmia, Iran; 2 Skin Diseases and Leishmaniasis Research Center, Isfahan University of Medical Sciences, Isfahan, Iran; 3 Basir Laboratory Research and Development Center, Basir Medical Diagnostic Laboratory, Isfahan, Iran; 4 Hojjatieh Medical Diagnostic Laboratory, Department of Medical Parasitology and Microbiology, Hojjatieh Hospital, Isfahan, Iran; 5 Nursing and Midwifery Care Research Center, Department of Operating Room, Isfahan University of Medical Sciences, Isfahan, Iran

## Letter to the editor

Sir,

Leishmaniasis is a parasitic infection caused by the Leishmania parasite, which is transmitted to humans through the bite of infected sand flies. The disease can cause a variety of symptoms, including disfiguring skin lesions and scars that may leave permanent psychosocial effects, such as declining mental health, social exclusion, and stigma [1]. 

Moreover, infected individuals are at a higher risk of suffering from lower quality of life, depression, anxiety, low body image, and loss of social status. There are several barriers to effective diagnosis and treatment of leishmaniasis, including poor health literacy, poverty, and limited access to healthcare in some regions. These factors can make it difficult for individuals to seek timely and appropriate care for the disease, which can lead to further complications and negative health outcomes [2].

Community-based interventions are an important strategy for the prevention and treatment of leishmaniasis. These interventions typically focus on reducing contact between humans and the sand fly vectors that transmit the disease, as well as controlling vector populations through the use of insecticides and other measures.

Protective management strategies, such as bed nets, can also be effective in reducing the risk of infection. In addition, efforts to reduce the reservoir host populations can help to limit the number of infected individuals.

Community participation is crucial in the success of these interventions, as it can help to increase compliance with preventive measures and promote awareness about the importance of seeking treatment. Education and training for health professionals, volunteers, and community members on screening and treatment methods can also play an important role in improving outcomes for individuals with leishmaniasis [3].

Community-based interventions can take many different forms, and may vary in terms of their content, design, and intended outcomes. These interventions are generally aimed at improving the health status and understanding of health issues among community residents, particularly those living in areas where leishmaniasis is endemic.

Ultimately, the effectiveness of community-based interventions will depend on a variety of factors, including the specific context in which they are implemented, the resources available, and the level of community engagement and participation. However, when designed and implemented effectively, these interventions can play an important role in reducing the burden of leishmaniasis and improving the health outcomes of affected individuals and communities [4]. 

Community-based interventions can take place in a variety of settings, including neighborhoods, workplaces, schools, religious institutions, charitable organizations, and government agencies. These interventions may be focused on mobilizing the public through a range of strategies, such as financial incentives, health education programs, information campaigns, and supportive groups.

In addition, efforts to improve access to health services and eliminate financial barriers can also play an important role in reducing the morbidity and mortality of leishmaniasis. Training and education activities can help to build the capacity of health professionals and community members to effectively diagnose and treat the disease, while also promoting preventive behaviors and early detection [5].

In conclusion, community-based interventions can be a critical component of efforts to prevent and treat leishmaniasis, particularly given the complex and multifaceted nature of the disease. Addressing the physical and psychosocial complications of leishmaniasis, such as scars, financial barriers, and healthcare accessibility, is essential for improving health outcomes and reducing the burden of the disease.

To be effective, community-based interventions must be designed with a holistic and comprehensive approach that takes into account the individual behaviors and social relationships within communities, as well as the broader political and economic context. This may involve working with local governments and other stakeholders to develop policies and strategies that promote effective prevention and treatment of leishmaniasis, as well as engaging with community members directly to promote awareness and education about the disease.

Ultimately, the success of community-based interventions will depend on a range of factors, including the level of community engagement and participation, the availability of resources and infrastructure, and the effectiveness of implementation strategies. However, when designed and implemented effectively, these interventions can play a critical role in improving health outcomes and reducing the burden of leishmaniasis in affected communities.

## Notes

### Competing interests

The authors declare that they have no competing interests.

## References

[1] Fernando SD, Siriwardana HV, Guneratne KA, Rajapaksa LC (2010). Some sociological aspects of cutaneous leishmaniasis in patients attending a tertiary referral centre in Colombo, Sri Lanka. Int Health.

[2] Pal B, Murti K, Siddiqui NA, Das P, Lal CS, Babu R, Rastogi MK, Pandey K (2017). Assessment of quality of life in patients with post kalaazar dermal leishmaniasis. Health Qual Life Outcomes.

[3] Ranawaka RR, Weerakoon HS, de Silva SH (2014). The quality of life of Sri Lankan patients with cutaneous leishmaniasis. Mymensingh Med J.

[4] Aghakhani N, Sharif Nia H, Samad Zadeh S, Toupchi V, Toupchi S, Rahbar N (2011). Quality of life during hemodialysis and study dialysis treatment in patients referred to teaching hospitals in Urmia-Iran in 2007. Caspian J Intern Med.

[5] Shiva F, Seifi N, Yaghoubi M, Momenzadeh A, Moradi AT, Darchini-Maragheh E (2019). Evaluation of Quality of Life, Anxiety and Depression in Children with Cutaneous Leishmaniasis. International Journal of Pediatrics.

